# Two Live Attenuated Vaccines against Recent Low–and Highly Pathogenic H7N9 Influenza Viruses Are Safe and Immunogenic in Ferrets

**DOI:** 10.3390/vaccines6040074

**Published:** 2018-11-01

**Authors:** Larisa Rudenko, Irina Kiseleva, Elena Krutikova, Ekaterina Stepanova, Irina Isakova-Sivak, Svetlana Donina, Andrey Rekstin, Maria Pisareva, Ekaterina Bazhenova, Tatiana Kotomina, Anastasia Katelnikova, Arman Muzhikyan, Valery Makarov, Erin Grace Sparrow, Guido Torelli

**Affiliations:** 1 Institute of Experimental Medicine, St. Petersburg 197376, Russia; vaccine@mail.ru (L.R.); irina.v.kiseleva@mail.ru (I.K.); krutikova.iem@mail.ru (E.K.); isakova.sivak@gmail.com (I.I.-S.); sveta.donina@gmail.com (S.D.); arekstin@yandex.ru (A.R.); maria.pisareva@influenza.spb.ru (M.P.); sonya.01.08@mail.ru (E.B.); tstretiak@gmail.com (T.K.); 2 Institute of Preclinical Research Ltd., St. Petersburg 188663, Russia; katelnikova.ae@doclinika.ru (A.K.); mancho1@yandex.ru (A.M.); makarov.vg@doclinika.ru (V.M.); 3 World Health Organization, 1211 Geneva, Switzerland; sparrowe@who.int (E.G.S.); torellig@who.int (G.T.)

**Keywords:** H7N9, avian influenza, pandemic threat, live attenuated influenza vaccine

## Abstract

Influenza H7N9 virus is a potentially pandemic subtype to which most people are immunologically naïve. To be better prepared for the potential occurrence of an H7N9 pandemic, in 2017 the World Health Organization recommended developing candidate vaccine viruses from two new H7N9 viruses, A/Guangdong/17SF003/2016 (A/GD) and A/Hong Kong/125/2017 (A/HK). This report describes the development of live attenuated influenza vaccine (LAIV) candidates against A/GD and A/HK viruses and study of their safety and immunogenicity in the ferret model in order to choose the most promising one for a phase I clinical trial. The A/HK-based vaccine candidate (A/17/HK) was developed by classical reassortment in eggs. The A/GD-based vaccine candidate (A/17/GD) was generated by reverse genetics. Ferrets were vaccinated with two doses of LAIV or phosphate-buffered saline. Both H7N9 LAIVs tested were safe for ferrets, as shown by absence of clinical signs, and by virological and histological data; they were immunogenic after a single vaccination. These results provide a compelling argument for further testing of these vaccines in volunteers. Since the A/HK virus represents the cluster that has caused the majority of human cases, and because the A/HK-based LAIV candidate was developed by classical reassortment, this is the preferred candidate for a phase I clinical trial.

## 1. Introduction

Since 1958, when the first human infection with a zoonotic influenza A virus was serologically confirmed [1], a number of different subtypes of animal and avian influenza A virus have infected humans [2]. The spread of these viruses in bird populations, the likelihood of reassortment with other influenza viruses of birds and mammals, the proven transmission from birds to humans, and the high case-fatality rate (CFR) among infected people have raised significant concerns for global public health. Many scientists from around the world believe that avian influenza viruses could cause the next devastating global pandemic [3,4].

Human infections with avian influenza H7N9 viruses were first reported to the World Health Organization (WHO) on 31 March 2013 [5]. Since then, 1567 H7N9 human cases have been confirmed in China, with a CFR of 39% [6].

Vaccination is an essential public health measure to control influenza. In recent years, WHO recommendations for the composition of influenza vaccines have included both seasonal human influenza viruses [7] and potentially pandemic strains, predominantly of bird origin [6]. WHO considers the development of candidate vaccine viruses to be an essential component of the overall global strategy for pandemic preparedness. Recently, WHO has proposed two new epidemiologically relevant candidate vaccine viruses, derived from A/Guangdong/17SF003/2016-like highly pathogenic avian influenza virus (HPAIV) and A/Hong Kong/125/2017-like low pathogenic avian influenza virus (LPAIV) [8]. Using an Influenza Risk Assessment Tool (IRAT), the Centers for Disease Control and Prevention (CDC) rated the H7N9 (A/Hong Kong/125/2017) virus as having the highest pandemic emergence and impact scores among all potentially pandemic viruses evaluated [9].

In the face of the pandemic threat, cold-adapted live attenuated influenza vaccine (LAIV) may have a number of advantages over inactivated influenza vaccine, because of its ability to stimulate a broader and longer-lasting cross-protective immune response [10]. Newly developed H7N9 LAIVs may be used as the first line of defense of all age groups in the case if the H7N9 pandemic occurs. LAIVs are currently licensed in Russia, the United States, Canada, Europe, Australia and India, and have been shown to be safe and effective [11,12].

Recently, phase I clinical trials of a number of potential pandemic LAIV candidates were successfully completed in Russia [13,14,15,16,17,18]. Candidate strains for LAIV are generated in Russia by classical reassortment of the epidemiologically relevant wild-type (WT) strain with a cold-adapted master donor virus (MDV), as described elsewhere [19,20]. The creation of a 6:2 reassortant, by replacement of the genes coding for the internal proteins of WT virus with the corresponding genes of the MDV, is a reliable and reproducible method of attenuating WT viruses. The internal MDV proteins ensure that the reassortant virus is safe, while the surface glycoproteins (hemagglutinin (HA) and neuraminidase (NA)) of the WT virus provide the targets for the immune response in the vaccinated host. 

Classical reassortment in eggs can be used to develop potential pandemic LAIV candidates based on LPAIVs. In regard to the risk associated with manipulation with HPAIVs and their pathogenicity to chick embryos, it can be reduced by using a reverse genetics technique for generation of 6:2 cold-adapted LAIV candidates.

In 2014 an A/17/Anhui/2013/61 (H7N9) LAIV candidate (A/17/AH) was developed on an A/Leningrad/134/17/57 (H2N2) (Len/17) MDV backbone [21] and tested in volunteers in a phase I trial that showed the vaccine to be safe, well tolerated and immunogenic [14]. However, recent H7N9 viruses have reacted less well with post-infection ferret antiserum raised against A/Anhui/1/2013 strain, suggesting that it is not protective against currently circulating viruses [8].

The current study aimed to develop LAIV candidates based on currently circulating low pathogenic (A/HK) and highly pathogenic (A/GD) H7N9 avian influenza viruses, and to confirm their safety and immunogenicity in preclinical studies in a ferret model, with a view to choosing the most promising one for a phase I clinical trial. The study took place under the umbrella of the WHO Global Action Plan to increase supply of pandemic influenza vaccine [22].

## 2. Materials and Methods

### 2.1. Viruses

A list of influenza viruses used in this study is shown in Table 1. (i) The Len/17 cold-adapted MDV; all rights on the Len/17 belong to the Institute of Experimental Medicine (IEM), St Petersburg, Russia. (ii) A/Hong Kong/125/2017 (H7N9) avian influenza virus (human isolate) was provided by CDC, Atlanta, GA, USA. (iii) A/17/HK LAIV candidate was developed by classical reassortment of A/HK LPAIV with Len/17 MDV in specific pathogen-free (SPF) eggs, as described elsewhere [19,20]. (iv) The A/17/GD LAIV candidate, based on A/GD HPAIV, was generated by reverse genetics. Plasmid DNAs encoding the polybasic cleavage site-deleted HA gene and intact NA gene of A/Guangdong/17FS003/2016 were provided by Dr. Othmar Engelhardt (National Institute for Biological Standards and Control (NIBSC), UK). Since the NA gene contained 292 K residue (N2 numbering), which is known to confer resistance to the NA inhibitor Oseltamivir, the K292R mutation was introduced by site-directed mutagenesis prior to virus rescue. The reverse genetics plasmids carrying Len/17 MDV genes were generated as described earlier [23]. The candidate live attenuated cold-adapted reassortant vaccine viruses, A/17/HK and A/17/GD LAIV candidates inherited HA and NA from the wild-type parental viruses and six internal genes (PB2, PB1, PA, NP, M and NS)—from MDV (genome composition 6:2). Vaccine candidates retained the major phenotypic characteristics (cold adaptation and temperature sensitivity) of the MDV. Full-length sequencing of internal genes of H7N9 LAIV candidates revealed identity of its attenuating mutations to those of MDV; no attenuating mutations have been lost in the internal genes during. Genes coding surface proteins of LAIV candidates were identical to those of wild-type parental viruses. Experimental series of monovalent H7N9 LAIVs based on these two vaccine viruses contained 10^7^ EID_50_/mL. (v) The A/17/Anhui/2013/61 (H7N9) LAIV candidate, A/17/AH [21], was developed in IEM by classical reassortment of A/Anhui/1/2013 (H7N9) LPAIV and Len/17 MDV in eggs.

All work with pathogenic H7N9 viruses was performed in a biosafety level 3 facility.

### 2.2. Ethics Statement

All work with ferrets was conducted in accordance with European Union legislation [24]. The animal use protocol was approved by the Local Bioethical Committee of the Institute of Preclinical Research Ltd (St. Petersburg, Russia) (ethical approval code No. 1.64/17 (23 October 2017) Protocol of Local Bioethical Committee of the Institute of Preclinical Research Ltd). All inoculations, nasal washes and blood sample collections were performed with the animal under short-term anesthesia induced by intramuscular injection of Zoletil 100, 12.5 mg/kg of body weight; every effort was made to minimize suffering. At the end of the study, animals were humanely euthanized according to IACUC guidelines.

### 2.3. Animals

Male ferrets (*Mustela putorius furo*), aged 5–6 months and weighing 0.7–1.1 kg at the beginning of the experiment, were supplied by Scientific-Production Organization House of Pharmacy JSC (St. Petersburg, Russia). They were prescreened by routine HAI test [25] to ensure that they were negative to circulating human influenza viruses and the viruses being tested.

### 2.4. Study Design

The study was a randomized, placebo-controlled study to evaluate the safety and immunogenicity of two H7N9 LAIV candidates in ferrets. Naïve ferrets were given the LAIV candidate intranasally, at a dose of 7.0 lg EID_50_/mL, divided between the two nostrils. The volume of inoculum was 1 mL for each ferret (0.5 mL per nostril). PBS was used as a control.

**Figure 1:**
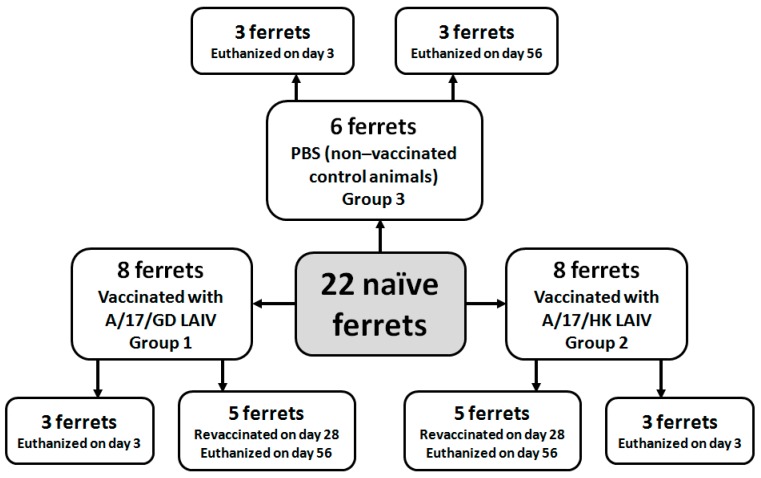
Experimental groups and study design.

On day 0, eight ferrets were immunized intranasally with A/17/GD LAIV candidate (group 1), eight ferrets were immunized intranasally with A/17/HK LAIV candidate (group 2), and six ferrets were given PBS (group 3) (Figure 1). On day 3, three ferrets from each group were euthanized and lung tissue samples were taken for histopathological analysis and virus detection. On day 28, the remaining five ferrets from groups 1 and 2 were revaccinated with 7.0 lg EID50/mL of A/17/GD or A/17/HK vaccine candidate, respectively. The remaining three ferrets from group 3 were re-inoculated with PBS. Nasal wash specimens were collected on days 1, 3 and 5 after each vaccine dose. Blood samples for serum preparation were collected 2 days before vaccination and on days 28 and 56. 

### 2.5. Clinical Signs and Morbidity Outcomes

Prior to infection, ferrets were randomly selected and housed individually. They were observed daily for clinical signs (body temperature, body weight, level of activity, nasal discharge, and sneezing). Nasal symptoms were scored as follows: 1—nasal rattling could be heard or the ferret sneezed during transport from its cage to the evaluation area; 2—there was evidence of nasal discharge on the external nares; 3—animals exhibited mouth breathing: 0—the animal exhibited none of these symptoms. Activity level was scored over a range from zero to three according to the extent that the animal could be induced to play: 0—the animal was fully playful; 1—the animal responded to play overtures but did not initiate any play activity; 2—the animal was alert but not at all playful; 3—the animal was neither playful nor alert. Scores were summed for each ferret, and group medians calculated.

Body temperature was measured using temperature data loggers (Star-Oddi, Reykjavik, Iceland) implanted into the peritoneal cavity.

### 2.6. Determination of Virus Load in Embryonated Chicken Eggs

Virus replication in the respiratory tract was assessed by endpoint titration of nasal washes and lung tissue samples in embryonated chicken eggs. 

All samples were analyzed by inoculation of 10-fold dilutions in 10–11-day-old embryonated chicken eggs and incubation at 32 °C for 72 h. The presence of influenza virus was detected by standard hemagglutination test with 1% chicken red blood cells (RBCs) as readout for positive eggs, as previously described [25].

### 2.7. PCR-Based H7N9 Vaccine Virus Detection

Nasal washes and lung tissue samples were also tested by real-time PCR for detection of influenza A virus RNA. RNA extraction from the nasal washes was performed using RIBO-sorb reagent kit for RNA/DNA isolation from human specimens (InterLabService, Central Research Institute of Epidemiology under Rospotrebnadzor, Moscow, Russia). Real-time PCR testing was performed using SuperScript III Platinum One-step qRT-PCR System (Invitrogen, Life Technologies Corporation, Carlsbad, CA, USA), as described elsewhere [26]. Primers and probes for the influenza A virus RNA amplification test with reagents were provided by CDC. To estimate the level of influenza virus RNA, a method of threshold cycle comparison was used. The RT-qPCR/mL titer was calculated in accordance with the method of Zang et al. [26] using Rotor-Gene 1.8.17.5 Software. AmpliSense primers and Taqman probes for influenza A virus M gene were used in a two-step RT-qPCR assay. The set values indicated for the two vaccines served as calibrators. The calibration line was constructed automatically (ROX channel) for 3 consequent 100× dilutions of the vaccine preparations in three replicates. The calculation was performed for each vaccine separately (R^2^ ≥ 0.98). The RT-qPCR titer per mL of nasal wash or lung suspension calculated for M gene target (H7N9) corresponds to the vaccine virus titer.

### 2.8. Hemagglutination Inhibition Assay

The influenza-specific antibody response in the non-vaccinated ferrets was measured by standard HAI test, as described elsewhere [25]. The serum antibody response after vaccination with H7N9 LAIV was assessed with 1.0% horse RBCs. Serum samples were pretreated with receptor-destroying enzyme (RDE) (Denka Seiken, Tokyo, Japan). A fourfold or higher rise in antibody titer after vaccination was considered a reliable indicator of seroconversion.

### 2.9. Determination of Influenza-Specific Ferret IgG/IgA

ELISA was used to measure H7 HA-specific serum IgG and IgA antibodies, as described elsewhere [27]. Whole purified H7N9 vaccine viruses, A/17/GD, A/17/HK and A/17/AH at 16 hemagglutinating units (HAU) per 0.05 mL, were used for absorption. Starting dilution for serum samples was 1:10, while for nasal wash specimens dilutions were prepared starting from 1:2. Anti-ferret IgG and IgA conjugates to horseradish peroxidase were supplied by Sigma (St. Louis, MO, USA).

ELISA titers were expressed as the inverse of the highest dilution that gave an optical density (OD) equal to or greater than twice the mean OD of the control (blank) wells. A fourfold or more rise in antibody titer after vaccination was considered a reliable indicator.

### 2.10. Necropsy

At the time of necropsy, a complete macroscopic post-mortem (gross pathology) examination was performed. The trachea and lungs were studied in detail, and the abdominal and pelvic cavities were examined. All lung lobes were inspected. Macroscopic changes in the lungs were scored according to color, the number of foci, and the severity of lesions. After necropsy, lungs were collected and weighed. Histopathological assessment of the lungs included such parameters as congestion, emphysema, hemorrhage, bronchioloalveolar hyperplasia and inflammation, and edema. 

### 2.11. Histopathology

Tissue sections of trachea and lungs were taken. One lobe of the lungs from each sacrificed animal was collected on day 3 post-vaccination and used for histological analysis; the other lobe was used for determination of viral replication. After fixation in 10% buffered formalin, lungs were embedded in paraffin and prepared for histopathological analysis. Tissue sections were stained with Alcian blue at pH 2.5, then with hematoxylin and eosin for microscopic studies to reveal goblet cells. A semiquantitative assay of respiratory tract tissue was performed to assess epithelial damage, inflammation and alveolar damage, scored as: 0 absent; 1 minimal; 2 slight; 3 moderate; 4 strong; and 5 severe [28].

### 2.12. Testing of Human Serum Samples

Human sera obtained from a phase I clinical trial of A/17/AH completed in 2015 (identifier no. NCT02480101 at ClinicalTrials.gov) was tested against newly recommended viruses, A/GD and A/HK in a standard HAI and MN tests [25]. Sera were collected after the first and the second dose of A/17/AH LAIV on day 28 and 56 post-vaccination [14].

### 2.13. Statistics

The Shapiro-Wilk test was used to assess distribution parameters (normality test). Differences between groups were analyzed statistically by one-way ANOVA, post-hoc Tukey test, and Kruskal-Wallis ANOVA by ranks, using StatSoft Statistica 10.0 (StatSoft Inc., Tulsa, OK, USA). Differences were considered significant at *p* ≤ 0.05.

## 3. Results

### 3.1. Clinical Observations in Ferrets

#### 3.1.1. Body Weight

**Figure 2:**
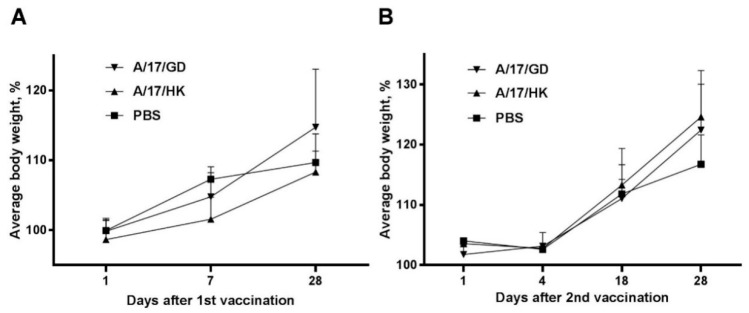
Changes in body weight of ferrets after administration of H7N9 LAIV expressed as % of initial body weight (mean + SEM): (**A**) First vaccination; (**B**) second vaccination.

The body weight of ferrets immunized with a single intranasal dose of H7N9 LAIV increased from day 1 to day 21 similar to the body weight increase in the control group treated with the phosphate-buffered saline mock vaccine (Figure 2A). There was no statistically significant difference between vaccinated and non-vaccinated animals (ANOVA, post-hoc Tukey test).

The initial body weight of ferrets before revaccination was 1.0 to 1.4 kg. Changes in body weight after the second vaccination are presented in Figure 2B. There was no statistically significant difference between the control group and the revaccinated animals after the second vaccination (ANOVA, post-hoc Tukey test).

Thus, vaccination and revaccination of ferrets with H7N9 LAIVs did not significantly affect body weight.

#### 3.1.2. Clinical Signs

**Figure 3:**
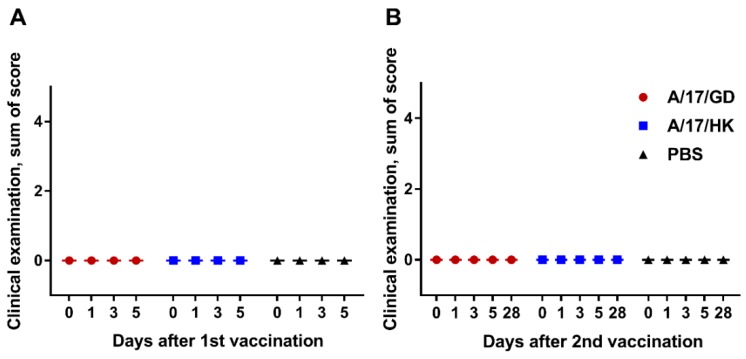
Clinical signs in ferrets after administration of H7N9 LAIV (group medians of sums of scores): (**A**) First vaccination; (**B**) second vaccination.

For the qualitative evaluation of a single administration of H7N9 LAIV, the group medians of sums of scores per ferret were calculated. As shown in Figure 3A, the vaccine had no effect on the general condition and behavior of ferrets on days 1–5 after vaccination (Kruskal–Wallis analysis, *p* > 0.05).

The results obtained after the second dose of H7N9 LAIV are presented in Figure 3B. Revaccination had no effect on the general condition and behavior of ferrets on days 28–56 (Kruskal–Wallis analysis, *p* > 0.05). 

There were no clinical signs of adverse effects in ferrets after one or two doses of H7N9 LAIV.

#### 3.1.3. Body Temperature

**Figure 4:**
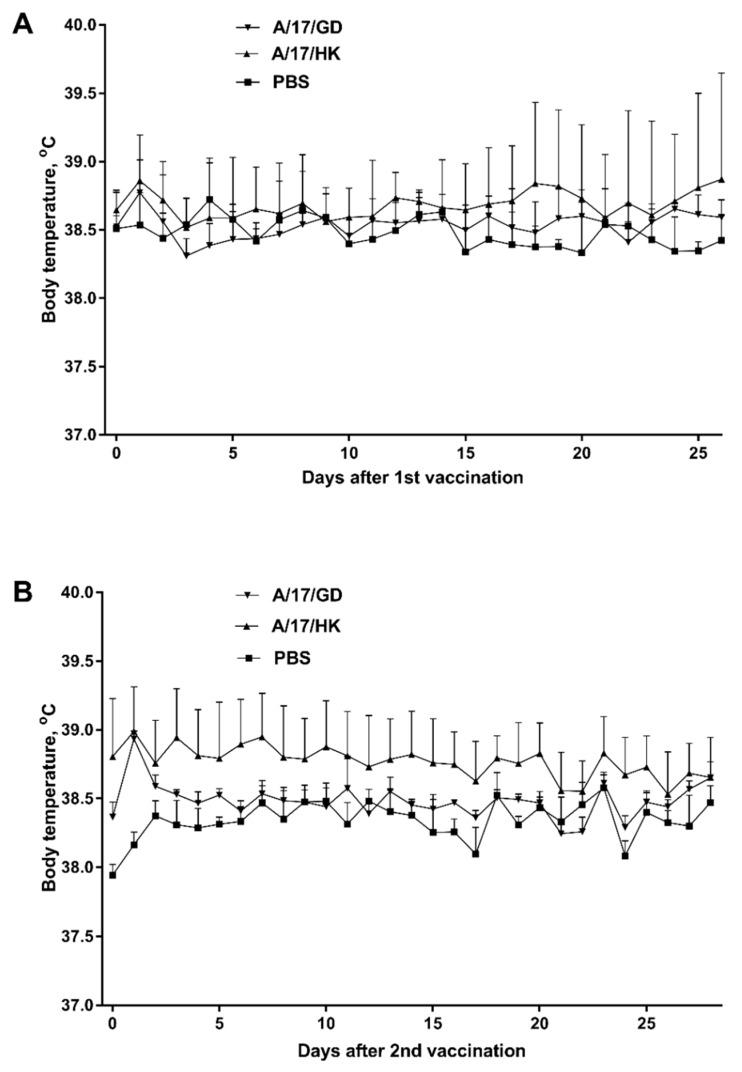
Body temperature of ferrets after vaccination with H7N9 LAIV (mean + SEM): (**A**) First vaccination; (**B**) second vaccination.

Univariate ANOVA showed that a single administration of vaccine had no effect on the ferrets’ body temperature (*p* > 0.05) on days 1–28 post vaccination (Figure 4A). Similarly, after revaccination, the ferrets’ body temperature was not significantly different from that of the control group on days 28–56 (*p* > 0.05, ANOVA) (Figure 4B).

### 3.2. Vaccine Virus Replication

Replication of A/17/GD and A/17/HK vaccine viruses in the upper respiratory tract of vaccinated animals was assessed by titration of nasal wash samples in embryonated chicken eggs on days 1, 3 and 5 after vaccination or revaccination. After the first dose, virus titers ranged from over 5 lg EID50/mL on day 1 to over 3 lg EID50/mL on day 5. There was no statistically significant difference between the two vaccine groups (Table 2). No infectious virus was found in lung tissue samples on day 3 after the first vaccination.

Notably, no live virus was found in nasal washes of ferrets in groups 1 and 2 after the second dose of H7N9 LAIV. None of the animals in groups 1–3 had live vaccine virus in the upper airways (Table 2). These data indicate that the single dose of H7N9 vaccine prevented vaccine virus replication after revaccination.

The presence of genetic material of H7N9 LAIV virus in the airways was also evaluated using real-time polymerase chain reaction (PCR). Results were similar to those obtained by titration of samples in eggs: H7N9 LAIV viruses were detected in nasal washes of vaccinated animals during the 5 days after vaccination (Table 2). Genetic material of the H7N9 LAIV viruses was also found in the lungs of vaccinated ferrets. Lung tissue samples of one ferret vaccinated with A/17/GD and of three ferrets vaccinated with A/17/HK were RNA-positive on day 3 (Table 2). 

On day 29 (one day after revaccination), genetic material of the vaccine virus was found in the nasal washes of one animal in each of the vaccinated groups (Table 2); this could be residual inoculum.

### 3.3. Pathomorphological Examination of Trachea and Lungs

To study morphological changes in the ferrets’ respiratory tract after a single dose of H7N9 LAIV, trachea and lung tissue were subjected to histological and semiquantitative analysis.

**Figure 5:**
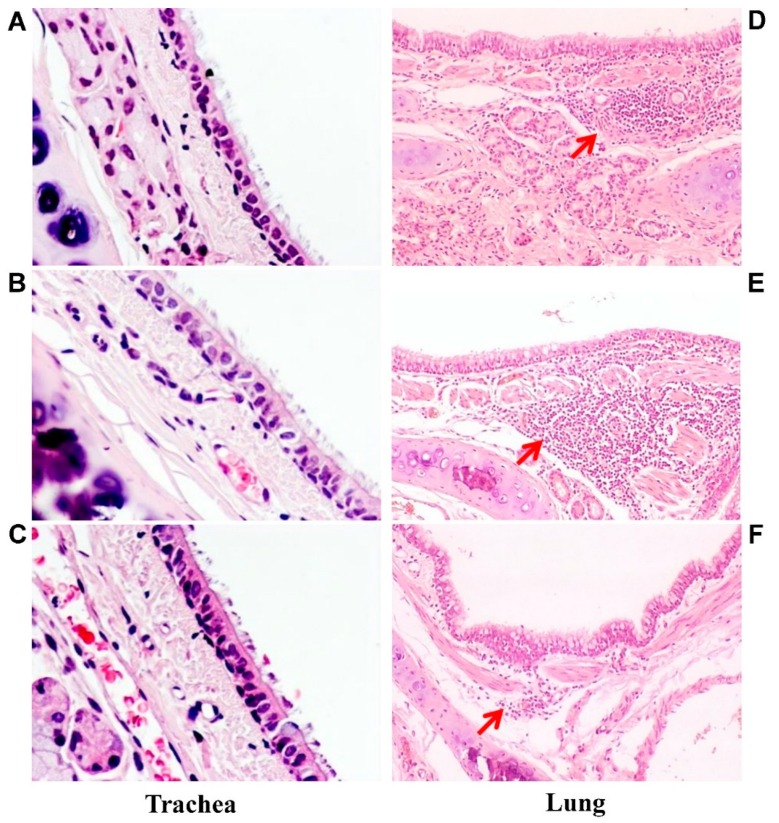
Microscopic analysis of trachea and lung pathology. Representative hematoxylin and eosin-stained trachea and lung sections of ferrets inoculated with one dose of A/17/GD LAIV (**A**,**D**), A/17/HK LAIV (**B**,**E**) or PBS (**C**,**F**): (**A**–**C**) trachea slices; magnification 200×. There are no pathological changes. (**D**–**F**) lung slices; magnification 100×. Red arrows indicate lymphocytic infiltration. (**D**) mild lymphocytic infiltration associated with hyperplasia of peribronchial lymphoid tissue. (**E**) moderate lymphocytic infiltration associated with hyperplasia of peribronchial lymphoid tissue. (**F**) mild lymphocytic infiltration associated with hyperplasia of peribronchial lymphoid tissue.

Macroscopically, there were no pathological changes in the trachea of any animals. The histological structure of the trachea of all examined animals was normal (Figure 5A–C).

Macroscopic analysis of the lungs of vaccinated animals also showed no pathological changes. Histological examination revealed mild nonspecific changes, in the form of hyperemia of the alveolar septa with small hemorrhages, minor lymphocytic and mononuclear infiltration of interstitial tissue, and weakly expressed focal peribronchitis accompanied by moderate hyperplasia of the bronchoalveolar epithelium and peribronchial lymphoid tissue (Figure 5D,E).

Specific morphological changes—the activation of bronchus-associated lymphoid tissue (BALT) characteristic of the development of an immune response to respiratory virus—were identified in groups 1 and 2 (Figure 5D,E).

Histological examination of the lungs of control ferrets (group 3) revealed mild nonspecific changes in the form of hyperemia of the alveolar septa with small hemorrhages and mild focal peribronchitis without signs of hyperplasia of peribronchial lymphoid tissue (Figure 5F).

A semiquantitative analysis of the revealed pathologies is presented in Table 3. Some slight to moderate pathologies were detected in groups 1 and 2. Statistical analysis with Kruskal–Wallis ANOVA by ranks did not reveal any statistically significant difference between the groups (H = 1.72, *p* = 0.42).

### 3.4. Antibody Response to H7N9 LAIV

**Figure 6:**
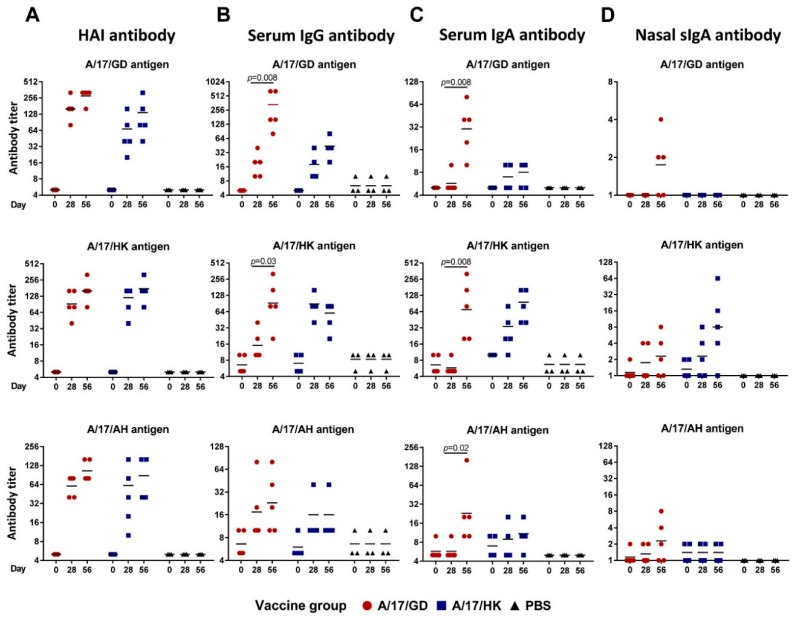
Homologous and heterologous antibody titers in ferrets given two doses of H7N9 LAIV intranasally at 28 days interval. Individual data and geometric means are shown. Test of Wilcoxon was used for comparison of antibody GMTs. (**A**) HAI antibody titers. (**B**) Serum IgG antibody titers. (**C**) Serum IgA antibody titers. (**D**) Secretory IgA antibody titers in nasal washes.

All ferrets were prescreened by routine hemagglutination inhibition test (HAI) to ensure that they were negative to circulating human influenza viruses and to the viruses being tested. None of the ferrets had HAI antibody titers ≥1:5 to any virus prior to vaccination. Both H7N9 LAIVs were highly immunogenic: 28 days after the first vaccination, all ferrets showed a 4-fold or greater increase in HAI antibody titers to homologous and heterologous H7N9 LAIV viruses (Figure 6). Homologous geometric mean titers (GMTs) were 176 for A/17/GD and 120 for A/17/HK. Both LAIVs induced cross-reactive HAI antibodies. The GMT of HAI antibody against A/17/HK and A/17/AH after one dose of A/17/GD reached 104 and 60, respectively. Similarly, the GMT of HAI antibody against A/17/GD and A/17/AH after one dose of A/17/HK reached 53 and 62, respectively.

After the second vaccination with H7N9 LAIV, the homologous HAI antibody titers increased slightly (to 279 and 160 for A/17/GD and A/17/HK, respectively), but no seroconversion was detected between the first and second dose (Figure 6A). The same pattern was noted with heterologous HAI antibodies.

Strikingly, the development of serum IgG and IgA titers was different for the two H7N9 LAIVs. For the majority of antigens tested, IgG antibody titers after two doses of A/17/GD vaccine were significantly higher than after a single dose, whereas A/17/HK LAIV induced high titers of IgG antibody after the first dose, and the second dose did not increase these antibodies (Figure 6B,C). These data suggest that two doses of A/17/GD might be required to establish fully functional antibody responses, while the A/17/HK vaccine is sufficiently immunogenic after a single dose. In addition, both H7N9 LAIVs induced homologous secretory IgA (sIgA) antibody in upper respiratory tract (Figure 6D). Although the A/17/HK vaccine was more immunogenic than the A/17/GD (GMT 8.0 versus 1.7 after the second dose), the difference didn’t reach statistical significance (*p* = 0.095, Mann-Whitney U test), most probably due to the low number of animals in the analysis. Interestingly, the A/17/HK-induced sIgA were strongly strain-specific, whereas two of five ferrets immunized with A/17/GD vaccine developed cross-reactive sIgA responses to heterologous H7N9 viruses (Figure 6D).

### 3.5. Heterologous Antibody Immune Response of Healthy Volunteers to H7N9 Viruses

In a separate experiment we investigated the cross-reactivity of human sera obtained from a phase I clinical trial of A/17/AH completed in 2015 against newly recommended viruses, A/GD and A/HK. A/17/AH LAIV-induced antibodies were poorly cross-reactive against recent H7N9 viruses (Table 4), confirming that there was no cross-protection by HAI or microneutralization (MN) tests in sera obtained from the earlier phase I clinical trial of A/17/AH to the newly recommended viruses. Therefore, new LAIV candidates are really needed.

## 4. Discussion

The threat of a new influenza pandemic is real. Currently, there is a large reservoir of influenza A viruses among animals and birds. A number of different subtypes of animal influenza A viruses have infected humans, including Hsw1N1 [29], H5N1 [6,30,31], H5N6 [32,33,34], H6N1 [35], H7N3 [36,37,38], H7N4 [39], H7N7 [40,41,42], H7N9 [5,43,44,45,46], H9N2 [6,47,48] and H10N8 [49,50].

One of the most important initiatives of the World Health Organization in preparing for a future influenza pandemic is focused on the development and evaluation of various vaccines against potentially pandemic influenza viruses. The presence of an effective vaccine at the beginning of a pandemic will largely determine the outcome of the first pandemic wave. Because of the inability to predict which strains will trigger subsequent pandemics, a large number of antigenically divergent variants representing various subtypes of potentially pandemic influenza viruses have been recommended for the development of pandemic vaccines [6,51]. Different approaches and platforms have been used to develop vaccines against potentially pandemic influenza viruses, and over the past few years, a lot of data has been accumulated on the safety and immunogenicity of these vaccines. The latest summary of clinical trials of pandemic vaccines can be found on the WHO website [52].

The effectiveness of preventive measures at the beginning of any influenza pandemic depends on the quality and availability of specific influenza vaccines. As the example of the 2009 pandemic showed, when a completely new strain emerged in circulation, against which relevant vaccines were not prepared, the most important action to mitigate the risk to public health is the rapid preparation, characterization and production of safe and immunogenic vaccines from an appropriate strain for immunizing high-risk groups. In this regard, LAIVs have a number of advantages over inactivated, the most important of which in the pandemic situation is the accelerated production of a large number of vaccine doses in short period of time. In addition, LAIV is administered by a painless intranasal route (nasal spray), which does not require the presence of qualified medical personnel for mass immunization, and also promotes the induction of cross-reactive factors in the adaptive immune response [53,54]. Another important advantage of LAIV is the induction of a mucosal immune response—the first barrier in the pathway of influenza infection, which significantly reduces the spread of the virus in the team [55]. In addition, the immunization with LAIV causes the formation of CD8 + CTL-immune response, which provides heterosubtypic defense of the organism [56,57]. All of these benefits were recognized by WHO experts, and LAIV was included in the WHO Global Action Plan to increase the supply of influenza vaccines [22], as well as to the WHO Global Plan for Pandemic Preparedness [51].

The current study was focused on the development and preclinical study of two H7N9 LAIV candidates generated on the base of recently isolated H7N9 viruses. These viruses are considered to be the most probable causative of the next influenza pandemic [9]. The first Russian H7N9 LAIV candidate was developed from an early isolate, A/Anhui/1/2013 [21] and tested in volunteers [14]. It was found to be well tolerated and safe and showed good immunogenicity [14]. However, as was shown in experiments with post-infection ferret anti-A/Anhui/1/2013 serum [8] and also in our experiment with human serum samples obtained in 2014-15 from a phase I clinical trial of A/17/AH LAIV, recent viruses react poorly with anti-A/Anhui/1/2013 antibody. Therefore, to be prepared for the potential occurrence of an H7N9 pandemic, two H7N9 LAIV candidates that are antigenically distant from A/Anhui/1/2013 (H7N9)-like viruses, A/17/GD and A/17/HK, were constructed on Len/17 backbone according to WHO recommendations [8].

The A/HK WT parental virus is a human isolate, for which the HA and NA have been antigenically and genetically characterized as representing the majority of novel currently circulating influenza A (H7N9) viruses [8]. A/HK is considered as a low pathogenic virus. Thus, the A/17/HK LAIV candidate was produced by classical reassortment in embryonated chicken eggs in a biosafety level 3 facility. The fifth H7N9 wave was characterized by the occurrence of a highly pathogenic form, as evidenced by the presence of a polybasic HA cleavage site. Therefore, to generate the A/17/GD LAIV candidate against this HPAIV the use of reverse genetics was required. In addition, reverse genetics technology allowed introducing an K292R amino acid change in the NA protein, thus generating an Oseltamivir-sensitive variant.

Both H7N9 LAIV candidates had the required vaccine genome composition of 6:2 and were tested to evaluate their safety, immunogenicity and protective efficacy in the ferret model. We were not able to challenge the ferrets with HPAIV because of the limitations of the animal facility. Thus, ferrets in groups 1 and 2 were revaccinated (challenged) with 7.0 lg EID50/mL of A/17/GD or A/17/HK vaccine candidate, respectively. In the absence of a challenge using wild type virus, protective efficacy of H7N9 LAIV candidates was evaluated by the absence of live vaccine virus replication post second vaccination dose. The use of attenuated influenza viruses as a surrogate measure for challenge with wild-type virus has been utilized in other studies, not only in ferrets [27], but also in human trials [58,59].

When ferrets were intranasally vaccinated or revaccinated with the H7N9 LAIV candidates they did not develop any clinical signs of influenza illness. Macroscopic and histological inspection of the trachea of ferrets inoculated with A/17/GD or A/17/HK did not reveal any effect of vaccination. Examination of the lungs of ferrets sacrificed three days after vaccination found only minor lesions similar to those seen in the control ferrets. Some slight to moderate pathological changes detected in the vaccinated ferrets could be associated with the development of an immune response.

Pronounced replication of vaccine viruses in the upper respiratory tract of ferrets after the first dose of vaccine was detected by titration of nasal washes in embryonated chicken eggs. Importantly, none of the ferrets had detectable replication of vaccine virus in the lungs. After the second dose, no live vaccine virus was found in nasal washes, indicating that one dose of H7N9 LAIV prevented vaccine virus replication after revaccination. These data suggest that a single administration of H7N9 LAIV might protect against corresponding variants of the WT H7N9 virus.

The presence of H7N9 LAIV viruses in the airways was also evaluated using real-time PCR. Genetic material of H7N9 LAIV viruses was found in the lungs of vaccinated ferrets on day 3 post-vaccination. After the second dose, genetic material of vaccine virus was found in the nasal washes of one ferret in each vaccinated group on day 29 (one day after revaccination); this may have been residual viral inoculum.

It should be noted that PCR detects the presence of viral RNA, including in material where virus replication does not occur, as well as RNA fragments and non-replicable virus particles. LAIV viruses can attach to the surface of lung epithelial cells, but are unable to replicate inside the cells because of their attenuated nature. Consequently, conclusions about the presence of viable virus can be made only on the basis of the virological method.

The absence of clinical signs of disease, the fact that virus replication was limited to the upper respiratory tract, and the minor pathological changes in ferrets’ airways confirmed the attenuated phenotype of the H7N9 LAIV candidates.

Administration of a single H7N9 vaccine dose induced high anti-HA antibody titers in ferrets. The second dose of vaccine did not further enhance the anti-HA antibody response, suggesting that a fully functional antibody immune response was established after a single dose. This protected the animals against the homologous LAIV virus replication after their revaccination given 28 days after the first vaccination. This finding is consistent with the results of other studies indicating that a single dose of A/Anhui/1/2013-based LAIV prevented virus replication in ferrets [22,60].

Nevertheless, the serum IgG and IgA antibody titers, as measured by enzyme-linked immunosorbent assay (ELISA), suggest that two doses of A/17/GD LAIV might be beneficial in the development of a strong immune response. The A/17/HK LAIV was sufficiently immunogenic after a single dose, and no boosting effect was seen after the second dose. Both vaccines induced cross-reactive HAI and IgG/IgA antibody titers; however, the faster induction of IgG and IgA antibody by the A/17/HK LAIV makes this the preferred candidate vaccine for further clinical development. 

In 2006, the WHO announced a Global pandemic influenza action plan for influenza vaccines (GAP). WHO, recognizing potential advantages of LAIV over the inactivated influenza vaccine in a pandemic situation, included LAIV in the GAP. Newly developed H7N9 LAIVs may be used as the first line of defense of all age groups in the case if the H7N9 pandemic occurs.

## 5. Conclusions

The two new live H7N9 influenza vaccines seem to be well tolerated and attenuated for ferrets. No clinical symptoms of respiratory disease were detected. A single intranasal administration induced a strong antibody immune response, protecting the animal from the homologous LAIV virus replication after the second administration. These results indicate that both H7N9 LAIV strains have the potential for use as a pandemic vaccine. However, since the A/HK virus represents the cluster that has caused the majority of human cases, and since it can be manufactured by classical reassortment, it is the preferred candidate for a phase I clinical trial in volunteers.

## Figures and Tables

**Table 1:** A list of viruses used in the study.

Virus	Genome Composition		Comment
	Surface Antigens HA and NA	Internal Protein Genes	
Len/17 (H2N2)	Len/17	Len/17	Master donor virus for LAIV
A/HK WT (H7N9)	A/HK WT	A/HK WT	Human isolate (avian influenza virus)
A/17/HK (H7N9)	A/HK WT	Len/17	LAIV reassortant virus obtained by classical reassortment in eggs
A/17/GD (H7N9)	A/GD WT modified *	Len/17	LAIV reassortant virus obtained by reverse genetics
A/17/AH (H7N9)	A/AH WT	Len/17	LAIV reassortant virus obtained by classical reassortment in eggs

* polybasic HA cleavage site deleted and K292R mutation introduced into NA protein; A/HK WT: A/Hong Kong/125/2017 (H7N9); A/GD WT: A/Guangdong/17FS003/2016 (H7N9); A/AH WT: A/Anhui/1/2013 (H7N9).

**Table 2:** Vaccine virus in lungs and nasal washes of ferrets, as measured by PCR and by culture in embryonated chicken eggs.

**Culture in Chicken Eggs (lg EID_50_/mL)**				
**Day after First Vaccination**	**A/17/HK**		**A/17/GD**	
	**Nasal Washes**	**Lung Tissue**	**Nasal Washes**	**Lung Tissue**
1	5.45 ± 0.243 (8/8)	n.d. ^1^	5.39 ± 0.242 (8/8)	n.d.
3	4.38 ± 0.161 (8/8)	< 1.5 ^2^ (0/3)	4.04 ± 0.254 (8/8)	<1.5 (0/3)
5	3.86 ± 0.289 (5/5)	n.d.	3.40 ± 0.391 (5/5)	n.d.
**Day after Second Vaccination**	**A/17/HK**		**A/17/GD**	
	**Nasal Washes**		**Nasal washes**	
1	<1.5 (0/5)		<1.5 (0/5)	
3	<1.5 (0/5)		<1.5 (0/5)	
5	<1.5 (0/5)		<1.5 (0/5)	
**PCR (RT–qPCR/mL)**				
**Day after First Vaccination**	**A/17/HK**		**A/17/GD**	
	**Nasal Washes**	**Lung Tissue**	**Nasal Washes**	**Lung Tissue**
1	5.690 ± 0.240 (8/8)	n.d. ^1^	5.731 ± 0.151 (8/8)	n.d.
3	4.239 ± 0.160 (8/8)	4.525 ± 0.625 (3/3)	4.817 ± 0.114 (8/8)	3.423 (1/3)
5	5.207 ± 0.254 (5/5)	n.d.	4.261 ± 0.681 (5/5)	n.d.
**Day after Second Vaccination**	**A/17/HK**		**A/17/GD**	
	**Nasal Washes**		**Nasal Washes**	
1	3.371 (1/5)		3.481 (1/5)	
3	<1.5 ^2^ (0/5)		<1.5 (0/5)	
5	<1.5 (0/5)		<1.5 (0/5)	

^1^ n.d.: not determined. ^2^ Estimated threshold limit value.

**Table 3:** Semiquantitative analysis of lung tissue and bronchial tree in male ferrets, day 3 post vaccination.

Histopathological Parameters	Score of Histopathological Changes								
	A/17/GD			A/17/HK			PBS		
	(Group 1)			(Group 2)			(Group 3)		
Animal index number	1	2	3	1	2	3	1	2	3
Exudate in lung lumen	0	0	0	0	0	0	0	0	0
Hypertrophy of bronchial epithelium	0	0	0	0	0	0	0	0	0
Hyperplasia of bronchial epithelium	0	0	0	1	0	0	1	0	0
Necrosis of bronchial epithelium	0	0	0	1	0	0	0	0	0
Exudate in bronchiole lumen	0	2	1	1	1	0	0	0	0
Hypertrophy of bronchoalveolar epithelium	0	0	0	1	0	0	1	0	0
Hyperplasia of bronchoalveolar epithelium	0	2	0	0	1	0	2	0	0
Necrosis of bronchoalveolar epithelium	0	0	0	0	0	0	0	0	0
Bronchitis	0	1	0	1	0	0	0	0	0
Peribronchitis	1	2	1	1	1	0	0	1	1
Bronchiolitis	0	2	2	1	0	1	0	0	0
Peribronchiolitis	0	1	1	1	0	0	1	0	1
Perivasculitis	0	1	0	1	1	1	0	1	1
Vasculitis	0	1	1	1	1	1	0	0	0
Interstitial infiltrate	0	1	1	1	1	1	0	0	0
Alveolitis	0	1	1	1	1	1	0	0	0
Hyperemia of alveolar septum	1	1	0	1	0	0	1	0	1
Alveolar emphysema	1	0	0	0	0	0	0	0	0
Alveolar hemorrhages	0	0	1	1	0	0	1	0	1
Total points per animal	3	15	9	14	7	5	7	2	5
Median	9			7			5		

0: no changes; 1: minimum changes; 2: moderate changes; 3: pronounced changes. Statistically significant differences were not found (Kruskal–Wallis test, *p* > 0.05).

**Table 4:** Homologous and heterologous antibody immune responses of healthy volunteers after vaccination with A/17/AH (H7N9) LAIV.

Assay	H7N9 Virus	After First Vaccination			After Second Vaccination		
		Seroconversion		GMT Rise	Seroconversion		GMT Rise
		No.	%		No.	%	
HAI	A/17/AH	3	10.3	1.7	19	65.5	3.4
	A/17/GD	1	3.4	1.1	1	3.4	1.3
	A/17/HK	0	0	1.0	0	0	1.0
MN	A/17/AH	14	48.0	3.4	21	72.4	5.5
	A/17/GD	2	6.9	1.3	7	24.1	1.6
	A/17/HK	n.d. ^1^	n.d.	n.d.	n.d.	n.d.	n.d.

^1^ n.d.: not determined.
